# A new species of the genus *Karschia* Walter, 1889 (Solifugae, Karschiidae) from China

**DOI:** 10.3897/zookeys.1269.180913

**Published:** 2026-02-13

**Authors:** Aidie Chen, Wenlong Fan, Feng Zhang

**Affiliations:** 1 Key Laboratory of Zoological Systematics and Application, College of Life Sciences, Hebei University, Baoding, Hebei 071002, China Hebei Basic Science Center for Biotic Interaction, Hebei University Baoding China; 2 Hebei Basic Science Center for Biotic Interaction, Hebei University, Baoding, Hebei 071002, China Key Laboratory of Zoological Systematics and Application, College of Life Sciences, Hebei University Baoding China

**Keywords:** Camel spider, mating behavior, morphology, solpugids, taxonomy

## Abstract

A new species of the genus *Karschia* Walter, 1889, Karschia (Karschia) baii**sp. nov**., is described from northwest China. We provide a detailed morphological description, diagnostic features, and illustrations for the new species, and present its known distribution in China. Additionally, the first photographs of wild mating for the family Karschiidae are presented.

## Introduction

The order Solifugae Sundevall, 1983 is one of the most fascinating yet least understood groups within the class Arachnida. They are mostly nocturnal arthropod predators, characterized by a pair of powerful, two-segmented chelicerae (commonly called “jaws”), voracious appetites, and extremely fast movement speeds (Punzo 1998). Solifuges are widely distributed in arid regions from tropical to temperate zones worldwide, where they are known by a variety of local names including camel spiders, false spiders, haarskeerders, roman spiders, sun spiders, and wind scorpions. Over the past century and more, researchers specializing in solifuge taxonomy and systematics have identified 15 families within this order, along with a variety of fossilized extinct lineages. Currently, this order comprises 13 families, over 150 genera, and approximately 1200 described species (Erdek 2025; WAC 2025). In China, solifuges are mainly distributed in the arid and semi-arid regions of the western part of the country. Prior to the commencement of this study, a total of four families, six genera, and 29 species of this group had been recorded in China (Harvey 2003; WAC 2025).

Karschiidae Kraepelin, 1899 is a small family within Solifugae, primarily distributed in arid and semi-desert regions of North Africa, Southern Europe, Western Asia, and Central Asia (Roewer 1934; Harvey 2003). The family currently comprises four genera: *Barrus* Roewer, 1933, *Barrussus* Roewer, 1928, *Eusimonia* Simon, 1879 and *Karschia* Walter, 1889, with a total of 56 valid species (WAC 2025). In China, Karschiidae is represented by two genera and 17 species: *Karschia* (15 species) and *Eusimonia* (2 species), mainly occurring in the arid and high-altitude regions of Xinjiang, Gansu, Qinghai, and Xizang (Fan et al. 2024; WAC 2025).

*Karschia* is the most diverse genus within Karschiidae, with its distribution center in Central Asia (Gromov 2004). The genus exhibits distinct morphological diagnostic characters: males possess a flagellum, composed of a long, spirally coiled seta; a cluster of elongated plumose modified setae (*pvd* setae) on the prolateral side of the cheliceral fixed finger; lack spiniform setae on the anterior margin of the propeltidium (Botero-Trujillo 2014); exhibit irregularly spaced, strongly differentiated teeth on the fixed finger (Roewer 1933; Gromov 2004; Bird et al. 2015); and females can be distinguished by a complex genital operculum showing pronounced intraspecific morphological variation (Gromov 2004). Harvey (2003) noted that this genus can be further divided into two subgenera: Karschia (Karschia) Walter, 1889 and Karschia (Rhinokarschia) Birula, 1935. Gromov (2004) concurred with this view and stated that these two subgenera can be distinguished by the presence or absence of a dorsal horn-like process on the male cheliceral fixed finger and the structure of the female genital operculum.

To further elucidate the diversity of *Karschia* in China, we have conducted systematic field surveys on solifuges in northwest China in recent years. A substantial number of *Karschia* specimens were collected, and through detailed morphological comparisons, a new species was identified. This paper describes and names the new species, providing new foundational data for the taxonomic revision of *Karschia* and for research on solifuge diversity in China.

## Material and methods

### Morphology

All specimens were collected by hand in 2025 from Gansu Province, China, and preserved in 75% and 95% ethanol. Observations, measurements, and photography were conducted under a Leica M205A stereomicroscope equipped with a DFC 550 CCD camera. Images were stacked using Helicon Focus ver. 8. Plate preparation and photo editing were performed using Adobe Photoshop 2020. Line drawings were made using INKSCAPE software (ver. 1.0.2.0). All specimens studied are deposited in the Museum of Hebei University (MHBU), Baoding, China.

The descriptive format primarily follows Birula (1938), with modifications based on Gromov (2004). Terminology for cheliceral chaetotaxy, dentition, and criteria for assessing primary homology of cheliceral teeth follow the system proposed by Bird et al. (2015). Leg setation and the use of the term “spiniform setae” adhere to the definitions of Botero-Trujillo (2014). Measurements and ratios were taken following the methodology of Fan et al. (2024).

### Abbreviations used in this study

**PL/PW**, the ratio of propeltidium length to width; **CH**, cheliceral height; **CL**, cheliceral length; **CW**, cheliceral width; ***fcp***, flagellar complex plumose setae; ***fcs***, flagellar complex subspiniform to spiniform setae; **FD**, fixed finger, distal tooth; **FM**, fixed finger, medial tooth; **FP**, fixed finger, proximal tooth; **FSD**, fixed finger, subdistal tooth/teeth; **FSM**, fixed finger, submedial tooth/teeth; **FST**, fixed finger, subterminal tooth/teeth; **FT**, fixed finger, terminal tooth; **MM**, movable finger, medial tooth; **MP**, movable finger, proximal tooth; ***mpd***, movable finger, prodorsal setae; ***mpm***, movable finger promedial setae; ***mpv***, movable finger, proventral setae; **MSM**, movable finger, submedial tooth/teeth; **MSP**, movable finger, subproximal tooth/teeth; **MST**, movable finger, subterminal teeth; ***pdp***, prodorsal proximal setae; **PF**, profondal teeth; **PFM**, profondal medial tooth; **PFP**, profondal proximal tooth; **PFSM**, profondal submedial tooth; **PFSP**, profondal subproximal tooth; **PH**, propeltidium height; **PL**, propeltidium length; ***pm***, promedial setae; ***pvd***, proventral distal setae; ***pvsd***, proventral subdistal setae; **PW**, propeltidium width; **RF**, retrofondal teeth; **RFA**, retrofondal apical tooth/teeth; **RFM**, retrofondal medial tooth; **RFP**, retrofondal proximal tooth; **RFSP**, retrofondal subproximal tooth/teeth; ***rlf***, retrolateral finger setae; ***rlm***, retrolateral manus setae; ***ric***, retrolateral interdigital condyle; ***rlpc***, retrolateral proximal setal cluster.

## Taxonomy

### Family Karschiidae Kraepelin, 1899

**Genus *Karschia* Walter, 1889**

#### Karschia (Karschia) baii sp. nov.

Taxon classificationAnimaliaSolifugaeKarschiidae

224648AD-C184-5202-98AA-8A057E795D23

https://zoobank.org/657C39BF-5E79-4791-985F-22EA84815BCD

Figs 1, 2, 3, 4, 5, 6, 7, 8, 9, 10, 11, 12, Table 1

##### Material examined.

***Holotype***: • ♂ (MHBU-Sol-2025-164-1), China: Gansu Province, Jiuquan City, Aksai Kazakh Autonomous County, 39.436788°N, 94.271840°E, altitude 2450.19 m, 15 May 2025, leg. Wenlong Fan, Haibin Zhang, and Chang Liu. ***Paratypes***: • 9♂♂ (MHBU-Sol-2025-164-2), 6♀♀ (MHBU-Sol-2025-164-3), same data as holotype; • 2♀♀ (MHBU-Sol-2019-12-32), CHINA: Gansu Province, Jiuquan City, Subei Mongol Autonomous County, Annanba Nature Reserve, Wushikate, 39.2361°N, 92.3392°E, altitude 2240 m, 17 July 2019, leg. Xinglong Bai; • 1♀ (MHBU-Sol-2019-21), China: Gansu Province, Jiuquan City, Subei Mongol Autonomous County, Dangchengwan, along Road X279, 39.6572°N, 95.1111°E, altitude 2798 m, 14 July 2019, leg. Xinglong Bai; • 1♀ (MHBU-Sol-2019-24), China: Gansu Province, Jiuquan City, Subei Mongol Autonomous County, Dangchengwan, Qingshandidao, 39.5775°N, 94.7494°E, altitude 2072 m, 13 July 2019, leg. Xinglong Bai.

**Figure 1.**
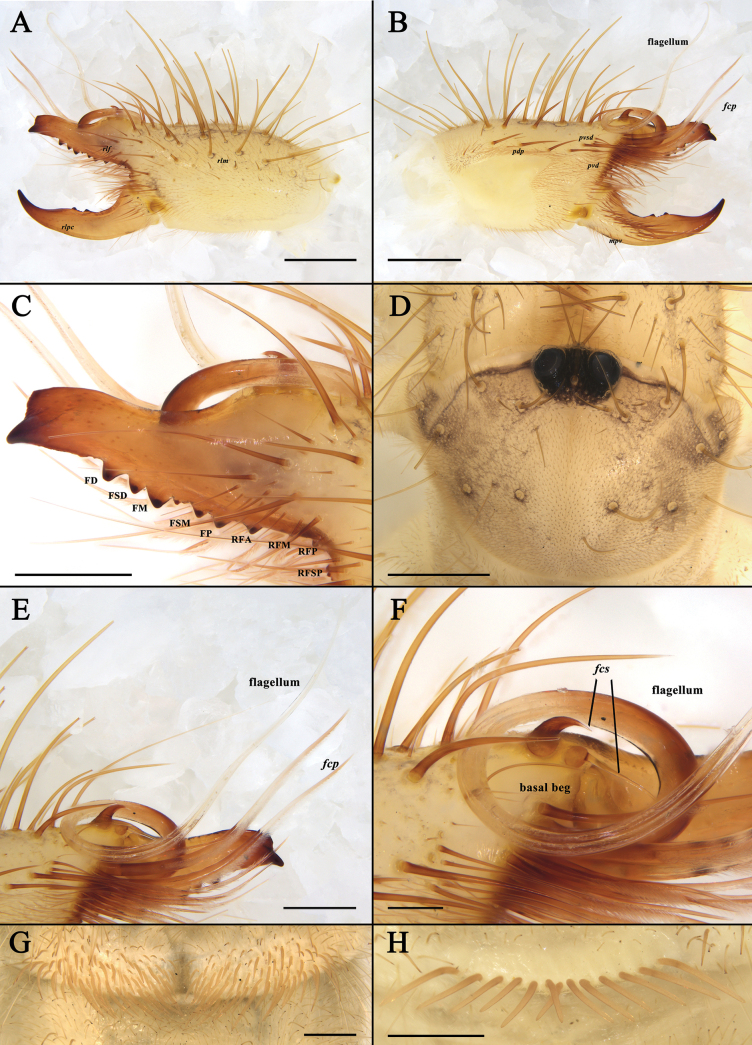
Male holotype of K. (K.) baii sp. nov. **A**. Left chelicera, retrolateral view; **B**. Left chelicera, prolateral view; **C**. Process on the fixed finger of chelicera, prolateral view; **D**. Propeltidium, dorsal view; **E**. Flagellar complex, prolateral view; **F**. Magnified view of the flagellar complex, prolateral view (taken from a different angle than **E**); **G**. Ctenidia on sternite III, ventral view; **H**. Ctenidia on sternite IV, ventral view. Scale bars: 1 mm (**A, B**); 0.5 mm (**C–F, H**); 0.2 mm (**G**).

**Figure 2.**
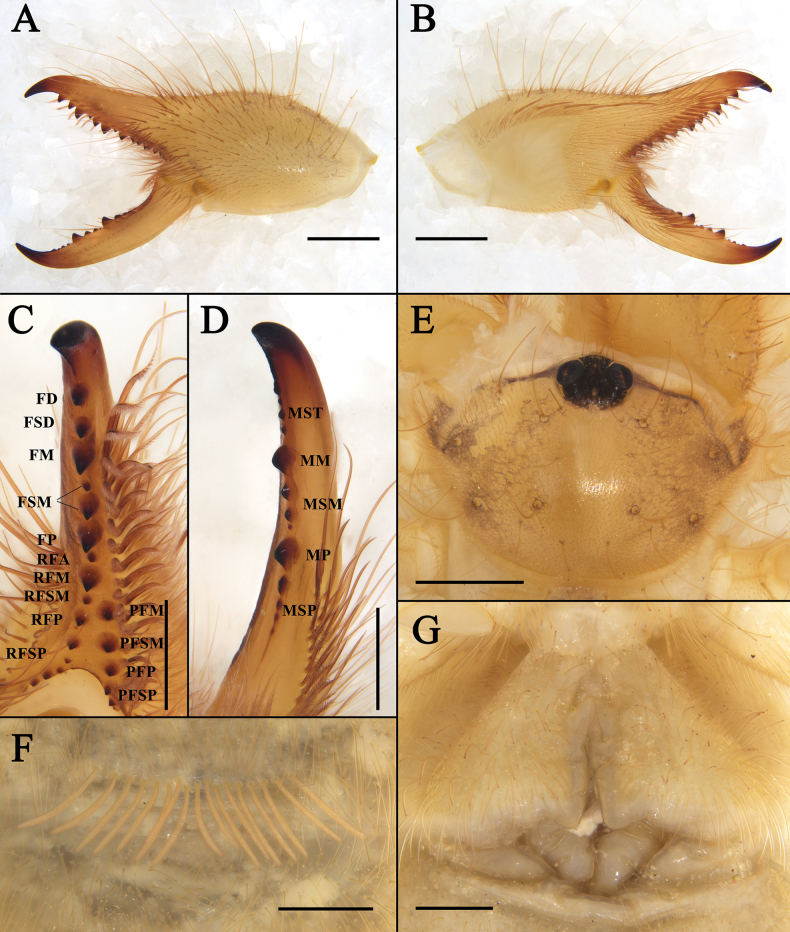
Female paratype of K. (K.) baii sp. nov., showing: **A**. Left chelicera in retrolateral view; **B**. Left chelicera in prolateral view; **C**. Dentition of the fixed finger; **D**. Dentition of the movable finger; **E**. Propeltidium in dorsal view; **F**. Ctenidia on sternite IV in ventral view; **G**. Genital operculum in ventral view. Scale bars: 1 mm (**A, B**); 0.5 mm (**C–G**).

**Figure 3.**
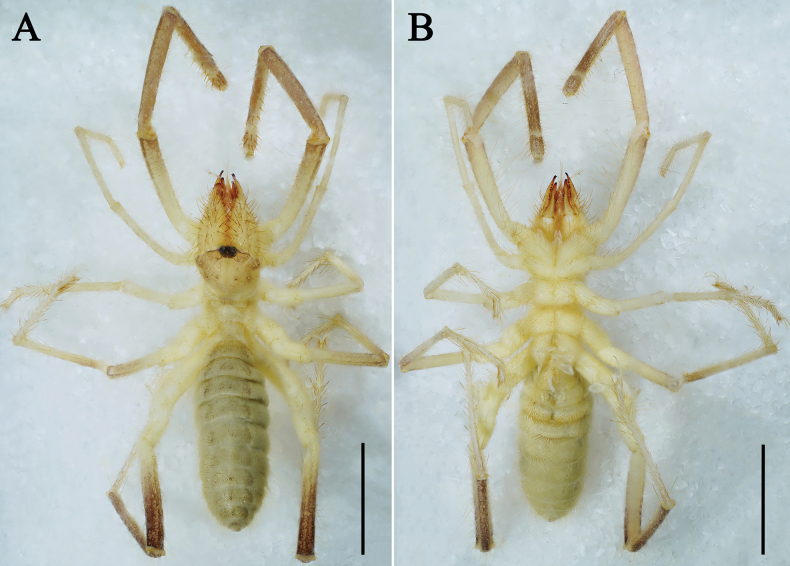
Karschia (Karschia) baii sp. nov., male holotype, general habitus. **A**. Dorsal view; **B**. Ventral view. Scale bars: 5 mm.

**Figure 4.**
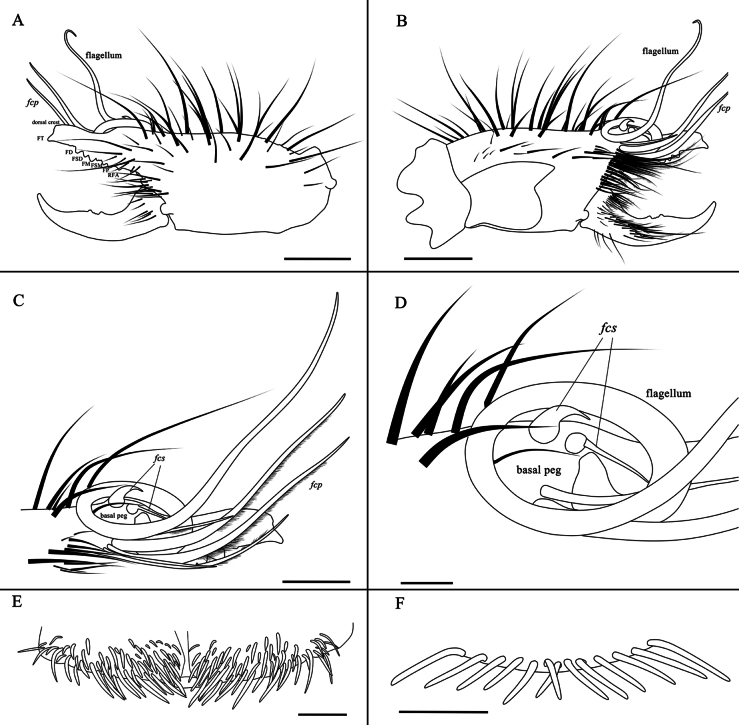
Drawings of the male K. (K.) baii sp. nov. **A**. Left chelicera, retrolateral view; **B**. Left chelicera, prolateral view; **C**. Flagellar complex, prolateral view; **D**. Magnified view of the flagellar complex, prolateral view (drawn from a different angle than **C**); **E**. Ctenidia on sternite III, ventral view; **F**. Ctenidia on sternite IV, ventral view. Scale bars: 1 mm (**A, B**); 0.5 mm (**C, D, F**); 0.2 mm (**E**).

**Figure 5.**
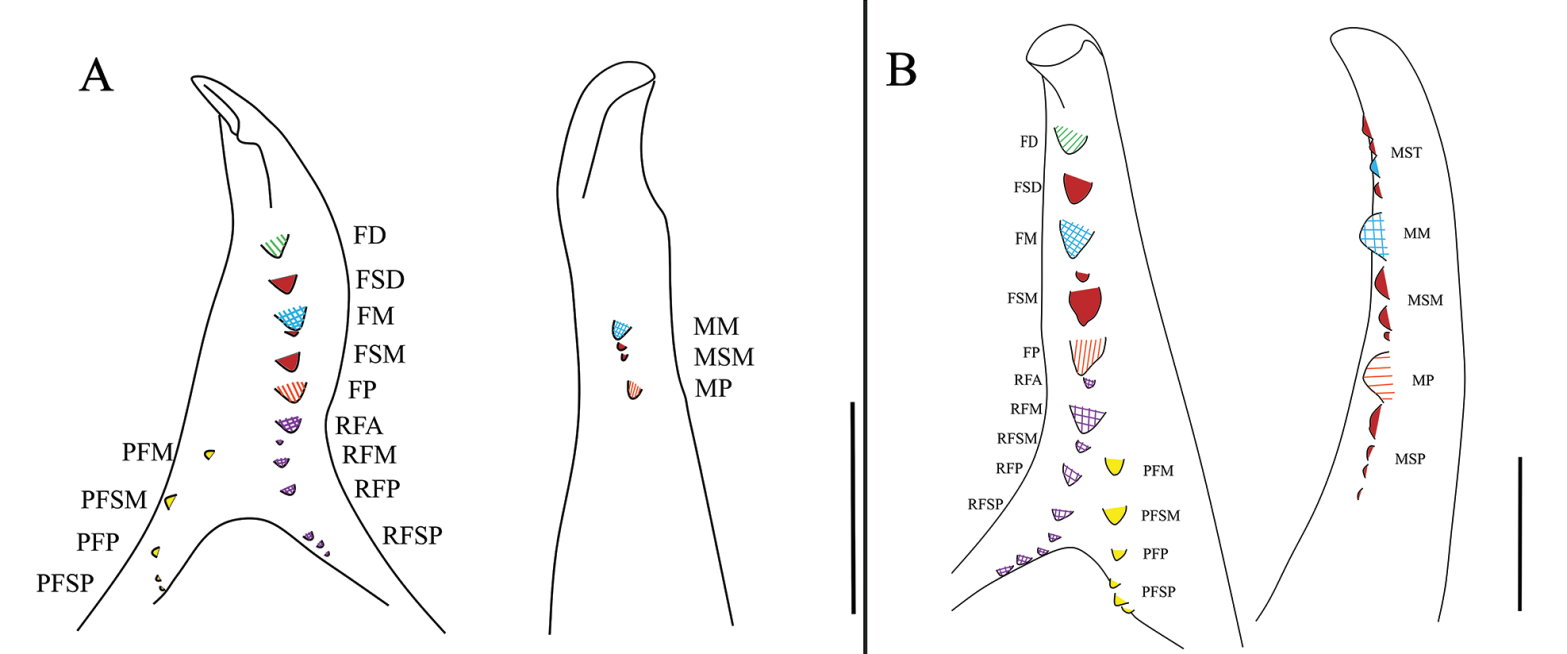
Drawings of the dentition of K. (K.) baii sp. nov. **A**. Male dentition, with the left image showing the dentition on the fixed finger and the right image on the movable finger; **B**. Female dentition, with the left image showing the dentition on the fixed finger and the right image on the movable finger. Scale bars: 0.5 mm.

**Figure 6.**
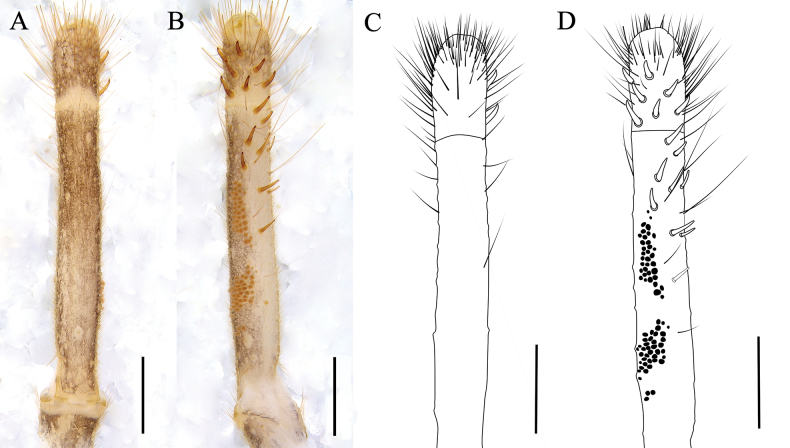
Left pedipalpal metatarsus and tarsus of the male K. (K.) baii sp. nov. **A**. Dorsal view; **B**. Ventral view; **C**. Dorsal drawing; **D**. Ventral drawing. Scale bars: 0.5 mm.

**Figure 7.**
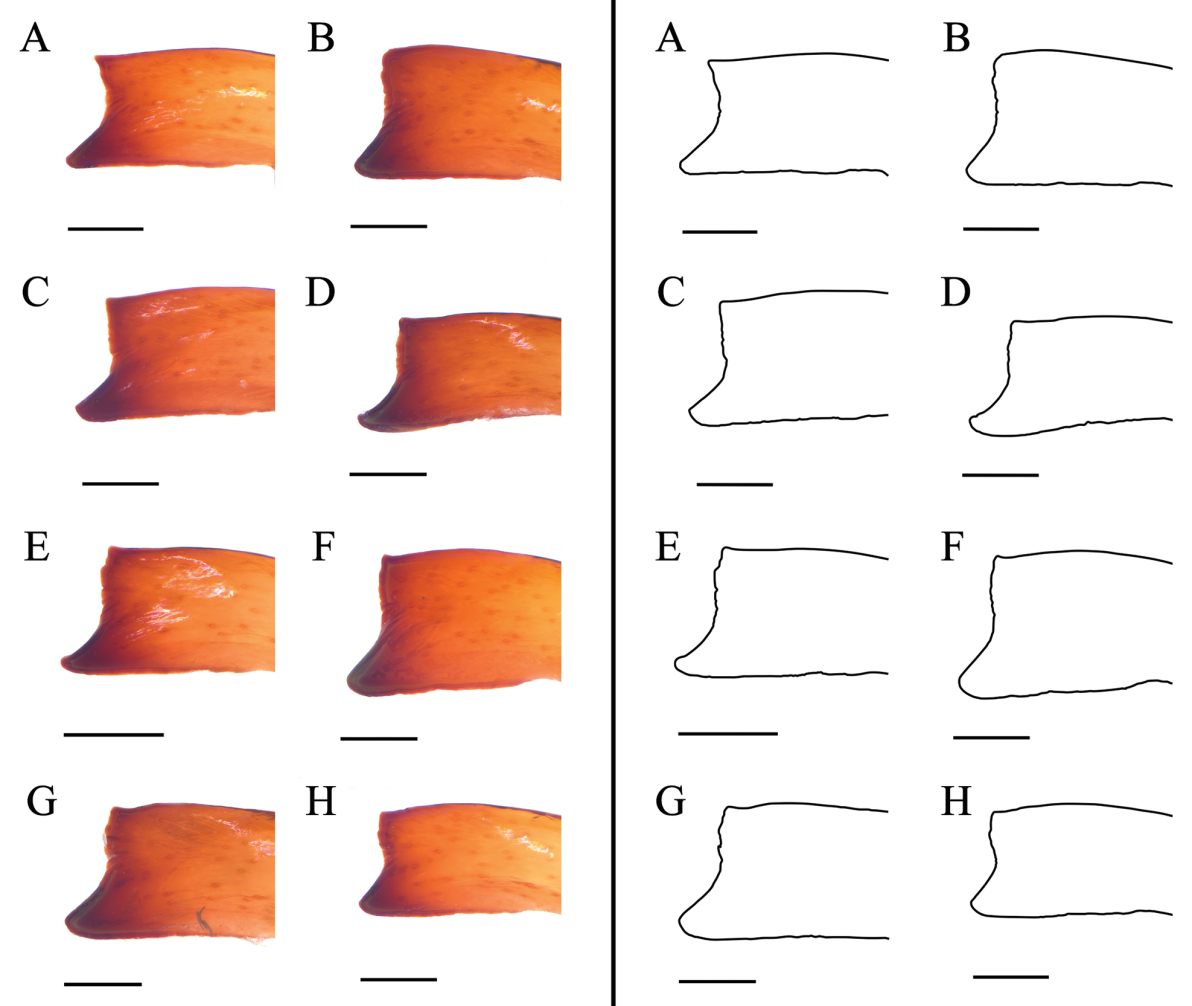
Left: dorsal bulge at the tip of the fixed finger of the male chelicera of K. (K.) baii sp. nov.; Right: corresponding line drawing. Scale bars: 0.2 mm.

**Figure 8.**
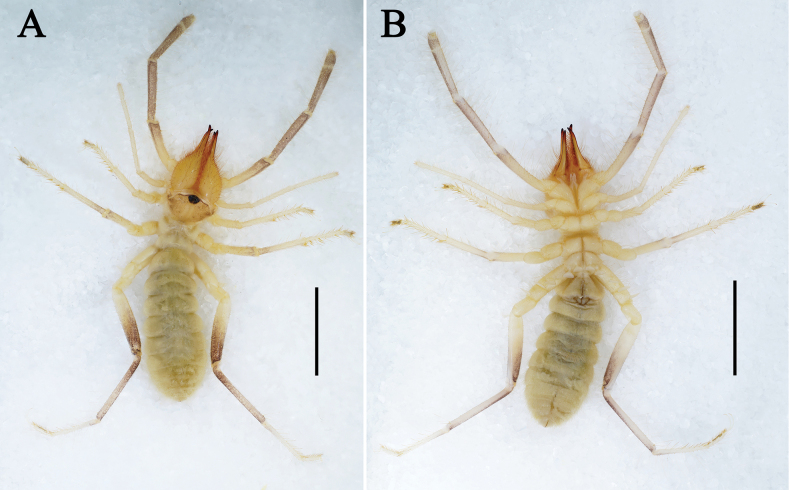
Karschia (Karschia) baii sp. nov., female paratype, general habitus. **A**. Dorsal view; **B**. Ventral view. Scale bars: 5 mm.

**Figure 9.**
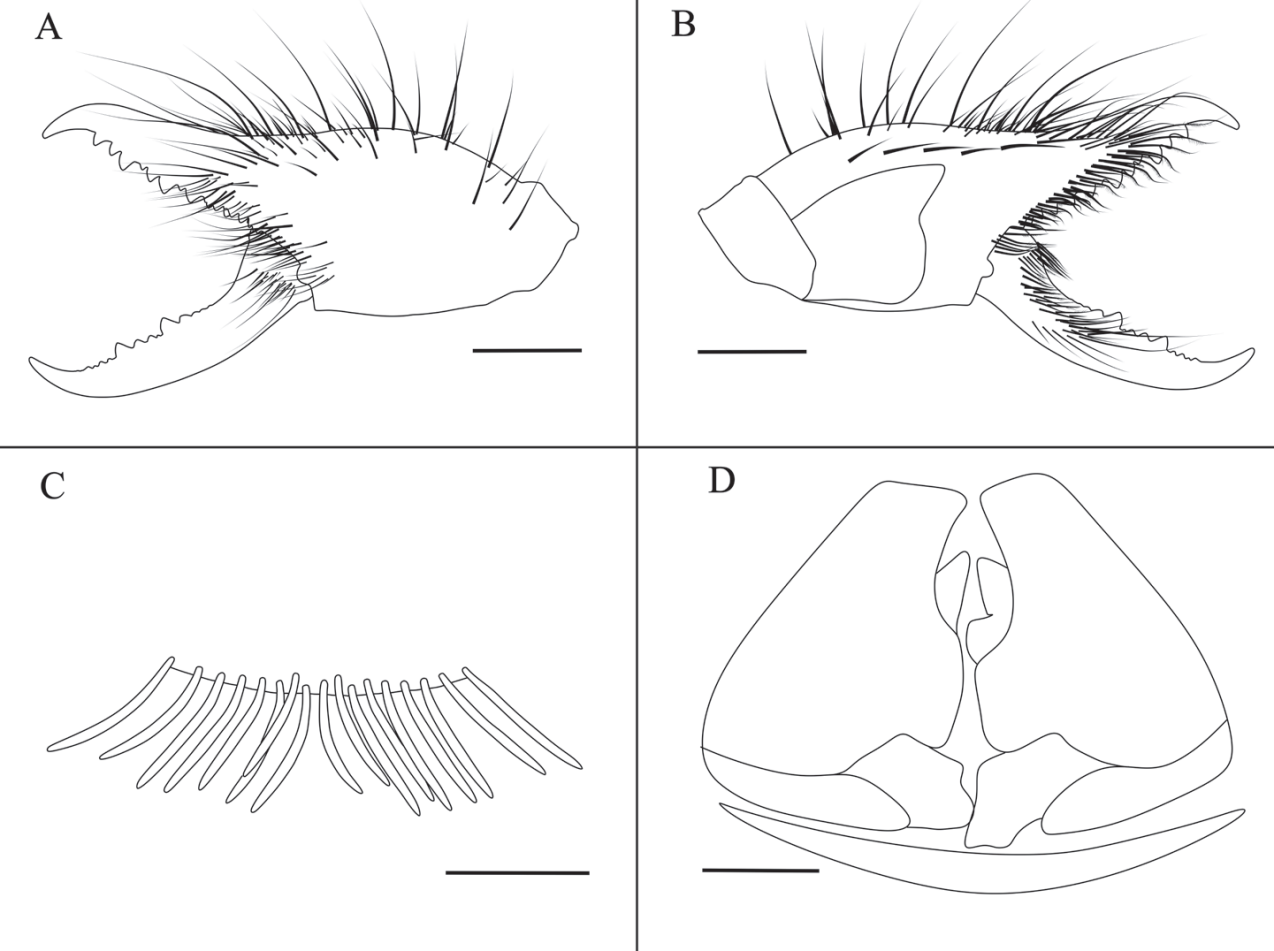
Drawings of the female K. (K.) baii sp. nov. **A**. Left chelicera in retrolateral view; **B**. Left chelicera in prolateral view; **C**. Ctenidia on sternite IV in ventral view; **D**. Genital operculum in ventral view. Scale bars: 1 mm (**A, B**); 0.5 mm (**C, D**).

**Figure 10.**
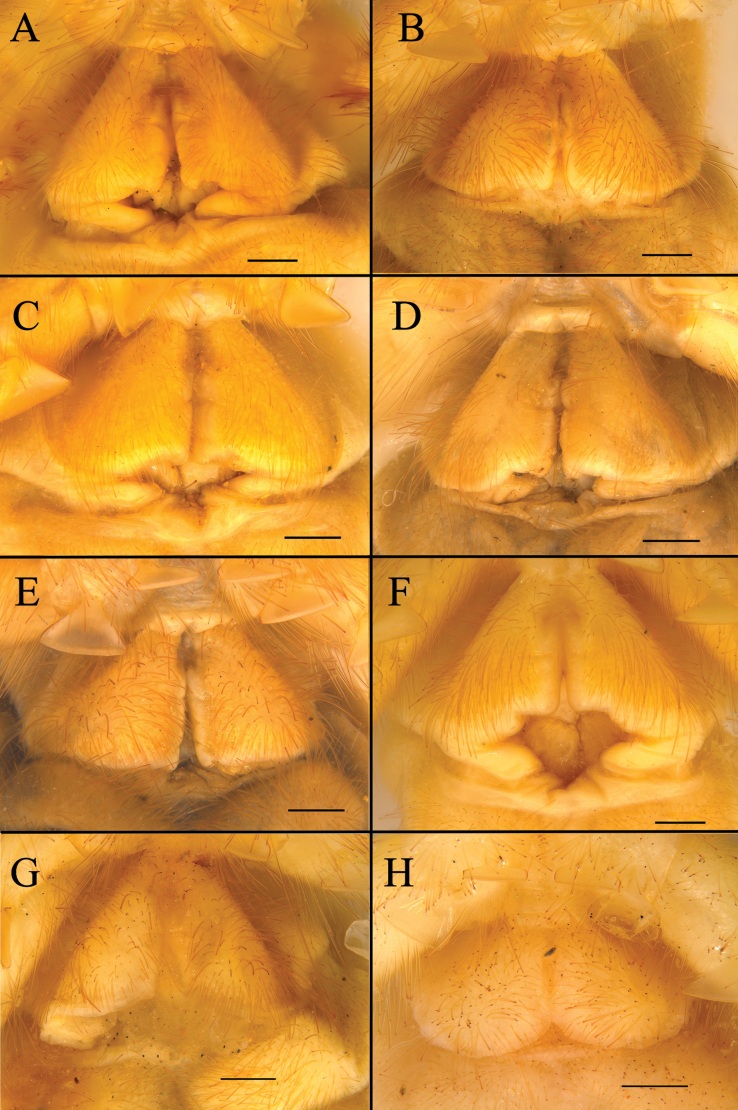
Genital operculum of female paratypes of K. (K.) baii sp. nov. Scale bars: 0.5 mm.

**Figure 11.**
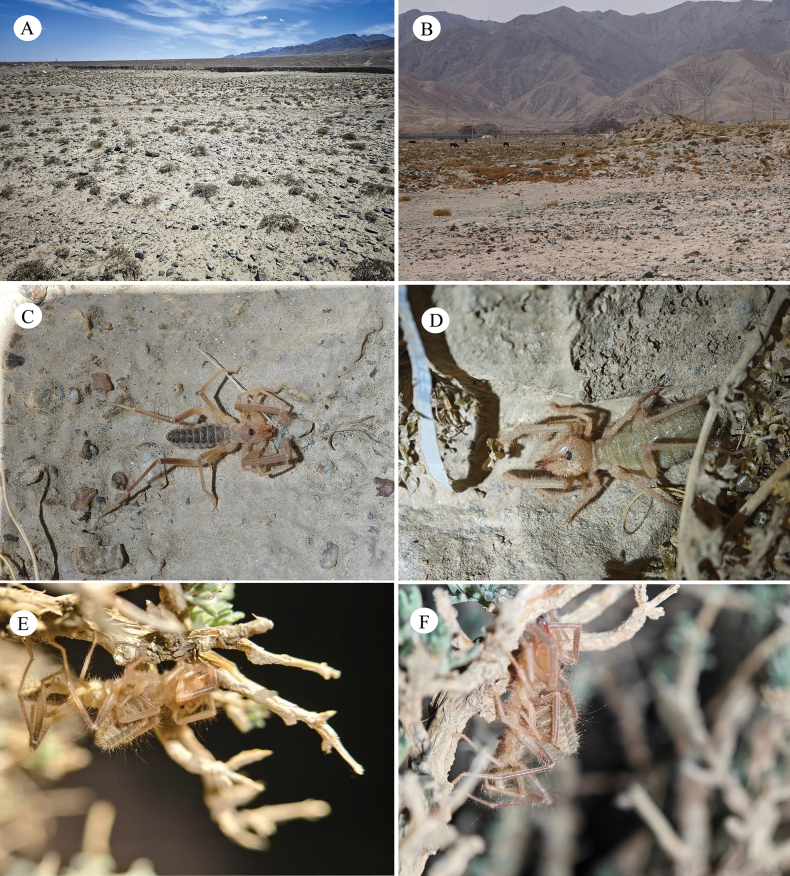
Habitat and living photos of Karschia (Karschia) baii sp. nov. **A, B**. Arid desert habitat with sparse vegetation in Gansu Province; **C**. Male individual in natural condition; **D**. Female individual in natural condition; **E, F**. Close-up of a mating pair on *Artemisia* branches, showing natural coloration and positioning during copulation.

**Figure 12.**
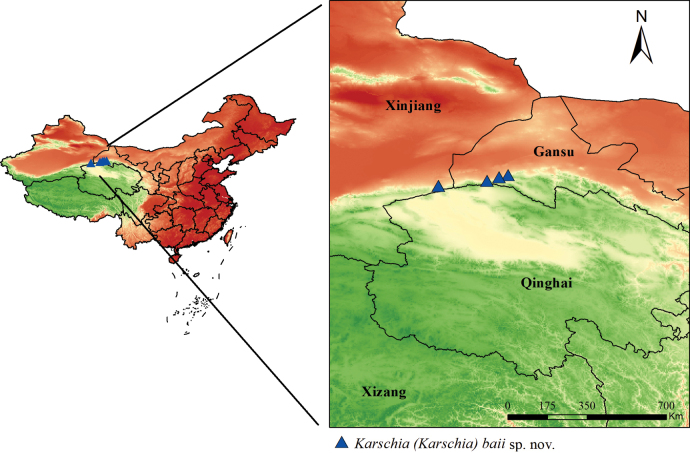
Known geographic distribution of K. (K.) baii sp. nov., showing the type locality in Gansu Province, China.

**Table 1.** Measurements (in mm) of male and female K. (K.) baii sp. nov.; L = length, W = width, H = height.

	Male holotype	Female paratype
Total body L (with chelicerae)	17.00	17.46
Propeltidium		
L	2.39	2.14
W	3.19	3.11
Chelicera		
L	4.29	4.79
W	1.48	1.72
H	1.42	1.44
Pedipalp total L	17.35	12.30
Femur L	4.51	3.38
Tibia L	5.34	3.58
Basitarsus L	3.83	2.76
Tarsus L	1.13	0.78
Leg I total L	13.51	9.83
Femur L	1.02	0.80
Patella L	3.47	1.94
Tibia L	3.78	2.75
Basitarsus L	2.07	1.63
Tarsus L	1.17	0.91
Leg II total L	12.14	8.11
Femur L	1.35	0.91
Patella L	2.51	1.43
Tibia L	3.45	2.30
Basitarsus L	2.10	1.58
Tarsus L	1.02	0.53
Leg III total L	16.15	11.11
Femur	1.66	1.20
Patella L	3.39	2.02
Tibia L	4.37	3.00
Basitarsus L	3.24	2.00
Tarsus L	0.97	0.56
Leg IV total L	24.27	16.99
Femur L	2.91	2.29
Patella L	5.80	3.84
Tibia L	5.86	4.21
Basitarsus L	4.53	2.99
Tarsus L	1.65	1.06

##### Etymology.

The species is named in honor of Dr Xinglong Bai (Museum of Hebei University), in gratitude for his generous contribution of numerous valuable specimens to this research.

##### Diagnosis.

The male of the new species possesses a coiled flagellum and lacks a horn-like process on the fixed finger, thus belonging to the subgenus Karschia (Karschia). It differs from all other *Karschia* species except Karschia (Karschia) latuis in the special dorsal crest of the male cheliceral fixed finger, but differs from K. (K.) latuis by the following characters (Fan et al. 2024): the dorsal crest above the tip of the fixed finger is higher and has a relatively smaller apex angle, whereas in K. (K.) latuis it is lower with a broader, more bluntly rounded apex (Fig. 1C); the flagellar complex subspiniform setae (*fcs*) are distinctly hook-shaped, while those of K. (K.) latuis are straighter and more gradually curved (Fig. 1F); the flagellum is longer (Fig. 1E); and the median longitudinal groove on the propeltidium is indistinct or weakly developed, contrasting with the prominent and deeply impressed groove in K. (K.) latuis (Fig. 1D). The female of the new species differs from K. (K.) latuis as follows: genital operculum more complex: overall outline of two isosceles right-angled triangles with right-angle vertices projecting anteriorly; distinct boundary between genital plates; small opening between inner margins on both sides; and posterior margin with transverse thickened structure and a pair of ventrally directed rod-like structures (Fig. 2G).

##### Description.

**Adult male (holotype and paratypes) (**Figs 1, 3–7).

***Measurements***. Metric data as shown in Table 1.

***Coloration*** (Fig. 3A, B). In specimens preserved in 95% ethanol: propeltidium predominantly yellowish-brown, median sulcus inconspicuous, yellowish; ocular tubercle black, surrounded by brownish spots; cheliceral manus predominantly yellow, covered with long yellowish-brown setae; fingers reddish-brown, especially on teeth; meso- and metapeltidium yellow; opisthosoma generally yellowish-gray, dorsally densely clothed with yellow setae; dorsal and ventral sclerites darker, dorsal sclerites with two dark longitudinal bands; pedipalps and legs yellow to brown, darkening from base to apex (most pronounced in pedipalps and leg IV); malleoli whitish.

***Propeltidium*** (Fig. 1D). Propeltidium wider than long (ratio of PL/PW 0.75), with long spiniform setae and dense short, fine setae perpendicular to surface; median sulcus inconspicuous; ocular tubercle with spiniform setae on each side, showing signs of shedding; interocular distance slightly less than eye length; propeltidium with median eyes only on ocular tubercle; no vestigial lateral eyes or pigmented spots (Bernard 1894; Miether and Dunlop 2016).

***Cheliceral dentition and processes*** (Fig. 5A). Fixed finger with median teeth series comprising three well-developed primary teeth (FP, FM and FD), graded as FM > FD > FP, plus two secondary FSM teeth and one secondary FSD tooth; secondary teeth distinctly smaller than primary teeth; fondal series consisting of profondal teeth series with five teeth (PFM, PFP, PFSM, two PFSP) and retrofondal teeth series with seven teeth (two RFA, RFM, RFP, three RFSP); fixed finger mucron bearing a specialized triangular dorsal crest (above the FT), apex slightly curved, hook-shaped, lacking subterminal (FST) tooth; movable finger with median teeth series bearing well-developed MM and MP teeth, subequal in size, and two secondary MSM teeth smaller than MM; no subproximal (MSP) or subterminal (MST) teeth present.

***Cheliceral setose areas and stridulatory plate*** (Fig. 1A, B). Retrolateral and dorsal surfaces of the manus with large, apically bifurcated setae (*rlm* series); retrolateral and dorsal surfaces of the fixed finger with simple apical setae of varying sizes (*rlf* series); *rlm* and *rlf* setae typically straight and stiff. Prolateral surface with an array of setal types: proventral distal setae (*pvd*) consisting of two rows of plumose setae extending to the level of the PFM tooth; proventral subdistal setae align in a single row, developing a well-defined comb-like structure (*pvsd* comb); promedial setae (*pm* series) short, brush-like, simple-tipped filiform setae covering carpet-like area; proximal prodorsal area with five long, thick, simple-tipped, unbarbed setae (*pdp*). Stridulatory plate extends longer than high, occupying most of the prolateral surface, bearing several *pdp* setae along dorsal margin; stridulatory ridge indistinct; movable finger prolateral surface bristles with dense setation; movable finger prodorsal setae (*mpd*) series as a slightly staggered row of plumose setae; adjacent movable finger promedial (*mpm*) and proventral (*mpv*) setal series tightly packed with fine, non-plumose setae.

***Cheliceral flagellar complex*** (Fig. 1B, E, F). The flagellar complex consists of flagellum, flagellar complex plumose (*fcp*) setae, and flagellar complex subspiniform (*fcs*) setae. Flagellum composite, rotatable, long, coiled, lacking lateral apophysis (defined here as a small protrusion on the proximal lateral side of the flagellum); attached to prodorsal surface of fixed finger via short stalk situated in deep alveolus. Flagellar complex plumose (*fcp*) setae (i.e., modified *pvd*) broadened and elongated, extending beyond distal tip of fixed finger. Flagellar complex subspiniform (*fcs*) setae, comprising two setae: one robust, gradually tapering; the other slender, basally swollen, abruptly narrowing to filiform, extending ventrally.

***Opisthosoma*** (Fig. 1G, H). Entire surface covered with nearly adpressed fine setae and numerous long, curved, bifurcate setae; tergites densely setose; ctenidia present on sternites III and IV (spiracular sternite III and post-spiracular sternite IV); sternite III with symmetric groups of needle-like ctenidia gradually increasing in size towards the posterior margin; sternite IV with single row of 13 slender, elongate ctenidia.

***Pedipalps*** (Fig. 6). All segments covered with short, fine setae and several long, thick setae; tarsus ventrally with seven short, strong spiniform setae arranged asymmetrically; no dorsal or lateral spines; metatarsus with eight ventral spiniform setae, 1/1/2/1/1/1/1 pattern (from distal to proximal); papillae dense, central region incomplete due to shedding during preservation.

***Legs***. Covered with numerous elongate setae and short, soft setae; elongate setae especially abundant on metatarsi; leg I lacking spiniform setae, with two small terminal claws; tibiae of legs II and III each with 3 ventral distal spiniform setae, while tibia of leg IV with 5; metatarsus with 9 (leg II), 10 (leg III), and 8 (leg IV) spiniform setae, respectively; tarsi anterior surfaces densely setose with short spiniform setae.

***Intraspecific variation in male fixed-finger morphology*** (Fig. 7). Observed in eight males of K. (K.) baii sp. nov., including holotype and paratypes; the overall shape and dentition of male cheliceral fixed finger consistent across all specimens; all individuals possess a distinct dorsal apical process, confirming stable diagnostic value; minor variation in length, extension, and apical angle of dorsal apical process, as well as in apex shape (ranging from acute to blunt) and contour of dorsal margin; apical angle ranges from 70° to 100°; process height (perpendicular distance from apex to dorsal margin of fixed finger) ranges from 0.16 to 0.22 mm; variation shows no correlation with collection locality or body size, indicative of non-genetic phenotypic plasticity, potentially ontogenetic or environmental in origin; all specimens share core diagnostic characters, supporting conspecificity.

**Adult female (paratypes)** (Figs 2, 5, 8–10).

***Measurements***. Metric data as shown in Table 1.

***Coloration*** (Fig. 8). In 95% ethanol, female similar to male in overall coloration; prosoma darker and opisthosoma paler, differences possibly reflecting variation in preservation conditions rather than intrinsic pigmentation.

***Propeltidium*** (Fig. 2E). Mostly like male, but female eyes smaller, and spiniform setae around ocular tubercle fewer in number and less robust than those of male.

***Cheliceral dentition and processes*** (Figs 2C, 2D, 5B, 9). Fixed finger with median teeth series comprising three well-developed primary teeth (FP, FM and FD); FD smaller, FM and FP similarly sized; plus three secondary teeth: one FSD and two FSM; all secondary teeth smaller than primary teeth. Fondal series comprising profondal teeth series with six teeth (PFM, PFP, PFSM, three PFSP), and retrofondal teeth series with nine teeth (RFA, RFM, RFP, RFSM, five RFSP). Mucron of fixed finger without subterminal (FST) teeth. Movable finger with median teeth series comprising two primary teeth (MM and MP), both similar in size; plus four MST, three MSM, four MSP; all secondary teeth upright and triangular in shape.

***Cheliceral setose areas*** (Fig. 2A, B). Generally similar to male, setose areas more slender and less developed in female; proventral distal setae (*pvd*) with single row of plumose setae; lacking flagellar complex.

***Opisthosoma*** (Fig. 2F, G). Entire surface covered with fine, soft setae and numerous dense, long setae; genital operculum triangular, with distinct demarcation between plates and a pair of clavate structures beneath posterior margin; genital opening situated beneath medial margin; sternite IV with single row of 17 slender, columnar ctenidia extending onto succeeding sternite.

***Pedipalps***. All segments covered with short, fine setae and long, thick setae; tarsus and metatarsus lack spiniform setae.

***Legs***. Same as in the male.

##### Intraspecific variation in female genital operculum.

(Fig. 10) Female paratypes exhibit a consistent genital operculum structure: an overall triangular outline, genital plates clearly demarcated with the right-angle vertex projecting anteriorly, and a transverse thickened ridge along the posterior margin. Minor intraspecific variation appears in the size and shape of the genital opening, ranging from slit-like to slightly elliptical; the width and contour of the posterior margin; the dimensions and contour of the genital plates, with measurements from mature specimens (excluding distinctly smaller juveniles) yielding lateral margins of 1.39 to 1.88 mm, inner margins of 1.16 to 1.38 mm, and posterior margins of 1.00 to 1.41 mm; and localized epidermal damage around the genital opening in some specimens. Erdek (2020) attributed such injuries and deformities to the female abdomen and genital region to contact with male tarsal setae and chelicerae during prey capture, coercive pre-copulatory behavior, and copulation. Observed differences likely result from male–female mating interactions. All variants fall within the diagnostic character range. This confirms the species as taxonomically stable.

##### Distribution and habitat.

(Figs 11A, 11B, 12) *Karschia* (*K.*) baii sp. nov. is currently known from Aksay Kazakh Autonomous County, Dangchengwan Town in Subei Mongol Autonomous County, and Wushikate in the Annanba Protected Area, all located in Jiuquan City, Gansu Province, China. Specimens were collected at elevations ranging from 2072 to 2798 m, with the lowest record from Qingshandidao near Dangchengwan (2072 m), the highest from along Road X279 near Dangchengwan (2798 m; paratype), and the holotype locality (Aksai County) at 2450.19 m.

All specimens were collected in sparsely vegetated sandy or sandy-gravelly habitats, commonly found on the surface or among low vegetation in open desert areas. The region is characterized by an arid climate with low precipitation, and the ground is covered primarily with coarse sand and gravel. Vegetation is sparse, composed of low, drought-tolerant desert plants, and occurs in scattered patches.

The elevational distribution of this species indicates that it inhabits mid- to high-elevation arid ecosystems in northwestern China. Notably, the genus *Karschia* exhibits an exceptionally broad altitudinal range in China, extending from approximately 1300 m in northwestern desert regions (Karschia (Rhinokarschia) liui) to as high as 4200 m in the alpine zones of the Tibetan Plateau (Karschia (Karschia) trisetalis). Karschia (Karschia) baii sp. nov. occupies the upper-middle segment of this elevational gradient and occurs at distinctly higher elevations than its congener species K. (K.) latuis. Together with its distinct morphological characters, this elevational differentiation provides additional support for recognizing K. (K.) baii sp. nov. as a valid new species.

## Discussion

We reported a new *Karschia* species from northwest China. This discovery increases the number of known *Karschia* species in China to 16, further enriching the documented diversity of solifuges in the country and providing important new material for taxonomic revisions, morphological evolution, and biogeographic studies of the genus *Karschia*. Notably, previous observations of solifuge mating behavior have been largely restricted to ground-level substrates or laboratory settings (Hrušková-Martišová et al. 2007; Hrušková‐Martišová et al. 2010; Rowsell and Cushing 2020; Peretti et al. 2021). In contrast, we observed mating behavior occurring at night on tree twigs (a rare arboreal reproductive habit for solifuges) and provided the first photographic documentation of this behavior (Fig. 11E, F). This suggests a more complex use of microhabitats during reproduction than previously assumed. Moreover, this finding further highlights the significance of northwestern China as a key center of solifuge diversity in Central Asia, underscoring the region’s unique value for biodiversity conservation and scientific research in arid and desert ecosystems.

## Supplementary Material

XML Treatment for Karschia (Karschia) baii

## Additional information

### Use of AI

No use of AI was reported.

### Author ORCIDs

Aidie Chen https://orcid.org/0009-0004-4611-342X

Wenlong Fan https://orcid.org/0009-0004-6854-6330

Feng Zhang https://orcid.org/0000-0002-3347-1031

### Data availability

All of the data that support the findings of this study are available in the main text.
